# Respiratory Sinus Arrhythmia Mechanisms in Young Obese Subjects

**DOI:** 10.3389/fnins.2020.00204

**Published:** 2020-03-11

**Authors:** Michal Javorka, Jana Krohova, Barbora Czippelova, Zuzana Turianikova, Nikoleta Mazgutova, Radovan Wiszt, Miriam Ciljakova, Dana Cernochova, Riccardo Pernice, Alessandro Busacca, Luca Faes

**Affiliations:** ^1^Department of Physiology and Biomedical Center Martin, Jessenius Faculty of Medicine, Comenius University, Martin, Slovakia; ^2^Department of Pediatrics, National Institute of Diabetes and Endocrinology, Lubochna, Slovakia; ^3^Department of Pediatrics, Jessenius Faculty of Medicine, Comenius University and University Hospital, Martin, Slovakia; ^4^Department of Engineering, University of Palermo, Palermo, Italy

**Keywords:** respiratory sinus arrhythmia, obesity, autonomic nervous system, information decomposition, multiscale analysis

## Abstract

Autonomic nervous system (ANS) activity and imbalance between its sympathetic and parasympathetic components are important factors contributing to the initiation and progression of many cardiovascular disorders related to obesity. The results on respiratory sinus arrhythmia (RSA) magnitude changes as a parasympathetic index were not straightforward in previous studies on young obese subjects. Considering the potentially unbalanced ANS regulation with impaired parasympathetic control in obese patients, the aim of this study was to compare the relative contribution of baroreflex and non-baroreflex (central) mechanisms to the origin of RSA in obese vs. control subjects. To this end, we applied a recently proposed information-theoretic methodology – partial information decomposition (PID) – to the time series of heart rate variability (HRV, computed from RR intervals in the ECG), systolic blood pressure (SBP) variability, and respiration (RESP) pattern measured in 29 obese and 29 age- and gender-matched non-obese adolescents and young adults monitored in the resting supine position and during postural and cognitive stress evoked by head-up tilt and mental arithmetic. PID was used to quantify the so-called unique information transferred from RESP to HRV and from SBP to HRV, reflecting, respectively, non-baroreflex and RESP-unrelated baroreflex HRV mechanisms, and the redundant information transferred from (RESP, SBP) to HRV, reflecting RESP-related baroreflex RSA mechanisms. Our results suggest that obesity is associated: (i) with blunted involvement of non-baroreflex RSA mechanisms, documented by the lower unique information transferred from RESP to HRV at rest; and (ii) with a reduced response to postural stress (but not to mental stress), documented by the lack of changes in the unique information transferred from RESP and SBP to HRV in obese subjects moving from supine to upright, and by a decreased redundant information transfer in obese compared to controls in the upright position. These findings were observed in the presence of an unchanged RSA magnitude measured as the high frequency (HF) power of HRV, thus suggesting that the changes in ANS imbalance related to obesity in adolescents and young adults are subtle and can be revealed by dissecting RSA mechanisms into its components during various challenges.

## Introduction

Obesity is a complex, multifactorial chronic disease associated with many adverse health consequences (Laederach-Hofmann et al., 2000; De Lorenzo et al., 2019). The prevalence of obesity in adults but also in children and adolescents prominently increased during last decades (World Health Organization [WHO], 2012). In the European Union, over 20% of school-age children (around 12 million children) suffer from overweight or obesity (Bagchi and Preuss, 2012). This results in an increasing occurrence of obesity-related complications (dyslipidemia, atherosclerotic changes, hypertension, impaired glucose tolerance, type 2 diabetes mellitus, etc.) even in childhood and adolescence (Vanderlei et al., 2010; Juonala et al., 2011; Cote et al., 2013; McCrindle, 2015; Ortega et al., 2016; Urbina et al., 2019).

Many cardiovascular disorders – including coronary artery disease, ventricular arrhythmia, arterial hypertension, left ventricular hypertrophy, and cardiomyopathy – are associated with obesity (Karason et al., 1999; Poirier et al., 2006). Autonomic nervous system (ANS) activity and imbalance between its two main components (parasympathetic and sympathetic nervous control) are important factors contributing to the initiation and progression of many cardiovascular disorders related to obesity (Ito et al., 2001; Cote et al., 2013; McCrindle, 2015; Ortega et al., 2016; Urbina et al., 2019).

To assess cardiovascular autonomic control changes in obese children and adolescents, heart rate variability (HRV) analysis in frequency domain was traditionally performed. High frequency (HF) HRV spectral power corresponding to the magnitude of respiratory-related heart rate oscillations – respiratory sinus arrhythmia (RSA) – was often analyzed due to its straightforward interpretation as an index of phasic parasympathetic activity, while the interpretation of slower oscillations in terms of sympathetic activity is more equivocal (Eckberg, 2000). Several studies demonstrated lower parasympathetic activity (lower HF HRV power) in obese children and adolescents (Paschoal et al., 2009; Thayer et al., 2010; Liao et al., 2014). In contrast, no significant differences in the HF power of HRV were observed in other studies between young obese subjects and healthy age- and gender-matched controls (Paschoal et al., 2009; Vanderlei et al., 2010; Javorka et al., 2016). Previous studies also demonstrated an impairment of arterial baroreflex (lower baroreflex sensitivity expressed as heart rate changes related to arterial blood pressure change) in obese children and adolescents, illustrating an impairment of reflex parasympathetic control (Honzikova et al., 2006; Krontoradova et al., 2008; Lazarova et al., 2009; Honzikova and Zavodna, 2016).

In order to shed light on the physiological mechanisms related to the controversial results reported above, this work undertakes a different approach than frequency domain analysis. Our motivation is the known fact that RSA results in humans from two principal pathways, reflecting a central mechanism (i.e., the connection of respiratory and cardiac control centers) and peripheral mechanisms (with a dominant role of high-pressure baroreflex mechanism). Although both these pathways are involved in the origin of RSA, their relative contribution varies with physiological conditions (Krohova et al., 2018). In this study, considering the potentially impaired parasympathetic control in young obese patients, our goal was to compare the relative contribution of baroreflex and non-baroreflex mechanisms in the origin of RSA in obese vs. control non-obese adolescents and young adults. To get insight into these mechanisms, we applied a recently developed information-theoretic approach to dissect causal interactions in multivariate time series, i.e., multiscale PID (Williams and Beer, 2010; Faes et al., 2017, 2018), computing the related measures on the cardiovascular and respiratory oscillations obtained at rest and during the application of two physiological stressors (i.e., orthostasis and cognitive load).

## Materials and Methods

The study group consisted of 58 adolescents and young adults, including 29 obese (O group) participants (14 female, age range: 12.4–22.7 years; median age: 15.4 years) and 29 age- and gender-matched healthy control (C group) subjects (age range: 12.5–22.1 years, median age: 15.8 years). The division to the O and C groups was based on the Cole’s chart (Cole et al., 2000), which takes age into account when the body mass index (BMI) is used to diagnose overweight or obesity. The majority of obese subjects (25 out of 29 participants) were in the range of BMI 29–38 kg/m^2^ corresponding to obesity classes I and II. The sample of subjects was recruited as a part of larger project focused on the study of obesity-related cardiovascular complications (e.g., see Czippelova et al., 2019). All measurements took place in the morning hours (from 8 am to 11 am), in a quiet examination room with temperature ranging between 22 and 25°C. All subjects must not suffer from any current or previous infectious disease (at least three weeks prior to the examination date), cardiovascular disease including hypertension (diagnosed using 24-h ambulatory blood pressure monitoring following examination), diabetes mellitus, psychiatric disorders, and hypothyroidism. All probands were instructed not to use substances influencing ANS or cardiovascular system activity during 24 h and not to perform strenuous physical activity during 48 h prior to examination. Fourteen female subjects in each group were examined in the proliferative phase (6th–13th day) of their menstrual cycle. All subjects or their legal representatives (in participants under 18 years of age) provided written informed consent to participate in the study. The study was approved by the Ethics Committee of Jessenius Faculty of Medicine, Comenius University. Detailed characteristics of obese and control groups are shown in Table 1.

**TABLE 1:** The main characteristics of participants.

	**Control group**	**Obese group**	***P*-value**
Age (years)	16.5 (2.6)	16.4 (2.7)	0.898
Height (cm)	170 (12)	171 (9)	0.881
Weight (kg)	61.3 (12.1)	96.7 (15.1)	<0.001
Body mass index (kg/m^2^)	21.0 (2.3)	33.2 (4.4)	<0.001
Fat mass (%)	18.7 (7.2)	38.7 (7.3)	<0.001
Skeletal muscle mass (kg)	27.81 (6.9)	33.31 (7.0)	0.004
Waist circumference (cm)	72 (7)	99 (12)	<0.001
Waist to hip ratio (–)	0.76 (0.05)	0.84 (0.09)	<0.001

*Values are expressed as mean (SD). The fat mass and skeletal muscle mass were evaluated using the InBody J10 device (Biospace, South Korea) which uses the direct segmental multi-frequency bioelectrical impedance analysis method (DSM-BIA). The differences between the groups of obese and healthy adolescents and young adults were evaluated by Mann–Whitney *U*-test, in addition to assessing the difference in body mass index that was evaluated using a *t*-test (with respect to data normality).*

In this work we used a subset of continuous recordings of ECG (horizontal bipolar thoracic lead; CardioFax ECG-9620, NihonKohden, Japan), finger arterial blood pressure (volume-clamp photoplethysmography method; Finometer Pro, FMS, Netherlands) and respiratory volume (respiratory inductive plethysmography; RespiTrace, NIMS, United States) measured during four phases of the study protocol: supine rest (15 min), head-up tilt (HUT) to 45 degrees for 8 min to evoke mild orthostatic stress, supine recovery (10 min) and non-verbal mental arithmetics (MA) in the supine position (6 min). As the next step, the 300 beats lasing segments of RR interval, the systolic blood pressure (SBP), and respiration volume signal (RESP) were extracted from the continuous recordings. For more detailed information about the protocol and time series extraction see Javorka et al. (2017) and Krohova et al. (2019).

### Data Analysis

As a first step, we calculated the spectral power of HRV in the HF band (0.15–0.4 Hz) using fast Fourier transform. The procedure started with resampling (cubic spline, 2 Hz) of the HRV time series to obtain an equidistant time series. Then, slower oscillations and trends were removed using the detrending procedure of Tarvainen et al. (2002). Subsequently, the mean power spectrum of the analyzed segment was computed and spectral power in the HF band was obtained by integration.

As a second step, we applied a recently proposed method, framed in information theory, to dissect causal interactions in multivariate time series according to the so-called PID (Williams and Beer, 2010; Faes et al., 2017, 2018; Krohova et al., 2019). PID was used in order to dissect the information transferred from SBP and RESP, considered as the sources of causal interactions, to the RR interval considered as the target, into contributions related to the information provided about the target individually by each source (interactions SBP→RR and RESP→RR) and the information provided as a result of the interaction between the two sources (interaction RESP→ SBP →RR). Specifically, PID decomposes the joint transfer entropy (TE) from (RESP, SBP) to RR evidencing the unique TEs representing information flowing from one source to the target that is not affected by the other source (measures U_RESP__→__RR_ and U_SBP__→__RR_), and the redundant TE (measure R_RESP,SBP__→__RR_) representing the amount of overlapped information from the two sources. PID enables also to separate redundant TE from the synergistic TE (S_RESP,SBP__→__RR_, related to the excess of information that two sources transfer to the target when they are considered together compared to the sum of the information transferred by both sources separately) – in this study analysis of synergy was not included in the results. The computation of these measures is based on a linear parametric modeling of the three time series which is described in detail elsewhere (Williams and Beer, 2010; Faes et al., 2017, 2018; Krohova et al., 2019).

From a physiological point of view, these measures represent various phenomena: the unique TE U_SBP__→__RR_ can be thought as reflecting the strength of the effects of SBP on RR unrelated to RESP occurring along the cardiac chronotropic baroreflex arm, while the unique TE U_RESP__→__RR_ represents the baroreflex-independent effect of RESP on RR [i.e., the non-baroreflex (mostly central) mechanism of RSA]. The redundant TE R_RESP,SBP__→__RR_ reflects the information transferred from RESP to RR through SBP (along the indirect pathway RESP→ < *SBP* < / →RR), thus describing baroreflex-mediated respiratory effects on heart rate.

Although in its original formulation PID analyzes the “raw” original time series measured from ECG, arterial pressure, and RESP signals, a recent development based on filtering the time series in order to eliminate the short temporal scales allows to compute the PID measures with reference to the slower oscillations (long time scales) contained in the observed processes (Williams and Beer, 2010; Faes et al., 2017, 2018; Krohova et al., 2019). Thus, while interactions between cardiovascular and respiratory time series are dominantly reflected at the short time scales (Faes et al., 2012; Javorka et al., 2017) included in the raw unfiltered time series, the advantage of multiscale PID is that all the above mentioned information measures could be calculated at any assigned time scale τ. In this study, in addition to raw time series analyzed at a time scale τ_1_ = 1 which includes all oscillations, we calculated PID measures also for a longer scale – τ_2_ determined – for each subject and experimental condition – as the time scale which removes the oscillations in the HF band and thus evidences slower oscillations [we refer to Krohova et al. (2019) for more detailed information].

### Statistical Analysis

Due to the non-normal distribution of the data the statistical comparison of a given measure (in both information and frequency domains) across conditions (supine rest, HUT, supine recovery, MA) for both time scales was performed using the non-parametric Friedman test with two *post hoc* pairwise comparisons using the Conover test: supine rest vs. HUT, and supine recovery vs. MA. The differences between the groups of obese and healthy adolescents and young adults were evaluated by means of the Mann–Whitney test for each measure of information decomposition on a scale representing original data (τ_1_) and slower oscillations (τ_2_), as well as for the spectral power of RR interval computed in the HF band. The results were considered statistically significant for *P*-values < 0.05. Results are reported in terms of *P*-values and effect sizes. Effect sizes were quantified by: Kendall’s coefficient of concordance *W* (comparison of supine rest vs. HUT, and supine recovery vs. MA) and by dividing the absolute (positive) standardized test statistic *Z* by the square root of the number of pairs (*n* = 58) (between group difference). According to Cohen’s classification of effect sizes, the value 0.1 represents small effect, 0.3 moderate effect, and 0.5 and above large effect.

## Results

### Respiratory Sinus Arrhythmia Magnitude

**FIGURE 1:**
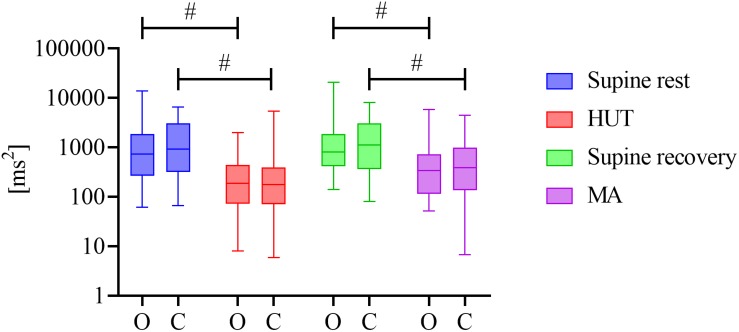
Distribution of HRV spectral power values in the HF band (*y*-axis with a logarithmic scale) over four phases (supine rest, HUT, supine recovery, and MA) for the groups of obese (O) and healthy (C) adolescents and young adults. The distributions are shown as box plots. # represents a statistically significant difference between preceding rest phase and physiological stress (orthostasis or mental arithmetic task).

Figure 1 reports the estimated magnitude of RSA, expressed as the distribution of the spectral power of HRV in the HF band computed in the two groups during the four phases of the experimental protocol. Both HUT and MA were accompanied by a significant decrease in the HF power of HRV (*P* < 0.001 for HUT and MA in O and C groups, effect size: 0.524–1). During the whole protocol we did not observe any significant difference in the RR interval spectral power between the two groups (0.460 ≤ *P* ≤ 0.692, effect size: 0.052–0.097).

### Effects of Stress Condition on the Interconnections Between Cardiovascular and Respiratory Signals

**FIGURE 2:**
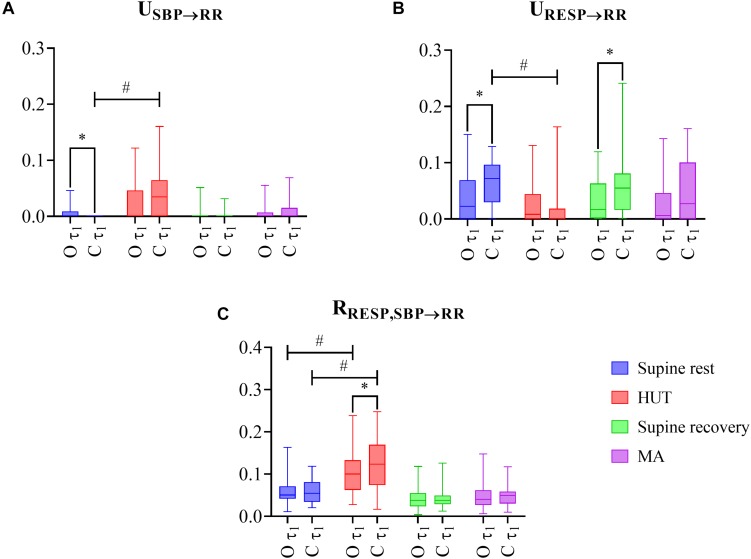
The results of multiscale information decomposition during four phases (rest, HUT, supine recovery, and MA) calculated for the raw (non-filtered) data (τ_1_) for the group of obese (O) and healthy non-obese control (C) adolescents and young adults. Graphs represent distribution of values in box plots for: **(A)** unique transfer entropy from SBP to RR (U_SBP__→__RR_) and **(B)** from RESP to RR (U_RESP__→__RR_), and **(C)** redundant transfer entropy (R_RESP,SBP__→__RR_) * indicates a statistically significant difference between the group of obese and healthy subjects and # represents a statistically significant difference between preceding rest phase and physiological stress (orthostasis or mental arithmetics task).

The distribution across subjects of the three considered PID measures computed on the raw data (without filtering, scale τ_1_ = 1) during the four phases of the protocol (supine rest, HUT, supine recovery, and MA) are shown in Figure 2 for both obese and control groups (O and C, respectively).

As the first step, we compared the impact of two types of physiological stress (supine rest vs. HUT, and supine recovery vs. MA) on the PID measures. For the C group, the transition from rest to HUT was associated with a significantly higher unique TE from SBP to RR (Figure 2A; *P* < 0.001, effect size: 0.655) and a significantly lower unique TE from RESP to RR (Figure 2B; *P* < 0.001, effect size: 0.596), while no significant changes were observed comparing MA with the previous rest period (U_SBP__→__RR_: *P* = 0.252, effect size: 0.029; U_RESP__→__RR_: *P* = 0.599, effect size: 0.001). For the O group, no significant changes across conditions were observed for either U_SBP__→__RR_ or U_RESP__→__RR_. On the other hand, the redundant TE R_RESP,SBP__→__RR_ was significantly higher during orthostasis in both groups (Figure 2C; *P* ≤ 0.001, effect size: 0.524–0.629).

As the second step, we evaluated the differences in the PID measures observed between the groups of obese and healthy subjects. The unique TE from SBP to RR was significantly higher in the O group compared to healthy controls (C group) at rest (Figure 2A; *P* = 0.004, effect size: 0.374). In contrast, the unique TE from RESP to RR was significantly lower in the obese group during both resting conditions (Figure 2B; *P* ≤ 0.049, effect size: 0.259–0.340). The redundant TE from RESP and SBP to RR was significantly lower during HUT in O group compared to controls (Figure 2C; *P* = 0.036, effect size: 0.275).

No significant between groups differences in PID measures were observed when only slower oscillations (τ_2_) were analyzed (*P* ≥ 0.179, effect size: 0.011–0.177, results not shown).

## Discussion

The major findings of our study include: (i) the observation of a well preserved parasympathetic nervous activity, expressed by RSA magnitude, and its responsiveness to stressors in young obese patients; (ii) the ability of PID to detect subtle abnormalities in RSA-related indexes in young obese patients compared to healthy controls, documented by reduced non-baroreflex respiratory effects on HRV (unique information transfer RESP→RR) in the resting condition and reduced baroreflex respiratory effects on HRV (redundant information transfer RESP→ < *SBP* < / →RR) during postural stress; and (iii) the ability of PID to reveal a reduced response to postural stress in young obese patients, documented by the lack of tilt-induced alterations of the cardiovascular and respiratory effects on HRV (unique information transfer RESP→RR and SBP→RR) compared with healthy controls.

The ANS plays an important role in the pathogenesis of cardiovascular disorders associated with obesity (Alam et al., 2009). The ANS is a very important control mechanism influencing energy balance and metabolic rate. Its activity is under the control of hypothalamic structures closely connected with the appetite control centers. Changes in the ANS activity and a dysbalance of its components can contribute to the obesity development but it is assumed that they are rather its consequence (Karason et al., 1999; Nagai and Moritani, 2004). A shift in cardiovascular autonomic control balance toward sympathetic nervous system dominance could contribute to the progression of serious cardiovascular complications in obese patients and significantly increase the risks of ventricular arrhythmia and sudden cardiac death in this population (Grassi et al., 1995; Muscelli et al., 1998). In previous studies, autonomic cardiovascular dysregulation in young obese patients was analyzed using linear and non-linear HRV analysis but the results of these studies were not consistent.

In accordance with several previous studies (Paschoal et al., 2009; Vanderlei et al., 2010; Javorka et al., 2016), no significant differences between young obese patients and controls were observed in this work in the RSA magnitude expressed as HRV HF power – an index reflexing the phasic cardiac parasympathetic activity. Our results extend the previous observations by the demonstration that HF power changes as a response to an application of two stressors (orthostatic test, MA) were similar in young obese patients and healthy controls. This finding indicates a well preserved parasympathetic nervous system reactivity in young obese subjects.

Applying PID analysis on the raw measured cardiovascular and respiratory time series, the orthostatic stress induced by HUT (but not the cognitive load induced by MA) resulted in an increased involvement of the high-pressure baroreflex as expressed by an increase in unique TE from SBP to RR in control group. This observation is in concert with the results of previous studies where the effect of orthostasis on the strength of the cardiac chronotropic baroreflex arm was analyzed in the frequency domain (Nollo et al., 2005) and using information-theoretic methods (Faes et al., 2013; Javorka et al., 2017). Higher baroreflex influence on heart rate was demonstrated also in both groups during orthostasis by an increase of the redundancy between respiratory and arterial pressure effects on HRV, indicating an increased importance of the indirect pathway RESP→ < *SBP* < / →RR during the unloading of baroreceptors associated with HUT. Moreover, considering the non-baroreflex mechanisms in the generation of RR intervals oscillations, their importance decreased during parasympathetic inhibition associated with orthostasis (decreased unique TE from RESP to RR during HUT in controls).

Although HF HRV power including its reactivity to physiological stressors was not able to distinguish between obese subjects and controls, the results of PID focused on disentangling basic mechanisms of RSA revealed some subtle between group differences. We applied multiscale PID to non-invasively assess the contribution of baroreflex (SBP→RR connection) and non-baroreflex (mostly central; RESP→RR connection) mechanisms to RSA. In our previous study, the relative contribution of these mechanisms was analyzed in young healthy subjects. At rest – both supine rest phase before HUT and recovery supine rest phase preceding MA – a lower contribution of non-baroreflex RSA mechanisms was found in the obese group, as reflected by the decreased unique TE from RESP to RR (U_RESP__→__RR_) in comparison with controls. This was accompanied by a slightly higher baroreflex contribution to RSA (U_SBP__→__RR_) at rest. This novel observation reveals the shift in the relative contribution of RSA mechanisms associated with obesity. Interestingly, this shift in RSA mechanisms is in the same direction as the shift observed during HUT (Krohova et al., 2018), probably mirroring a shift of the sympathovagal balance toward sympathetic activation and vagal withdrawal.

In response to the physical stress, another between-groups difference was detected: the orthostatic load was connected with a significantly lower redundancy between influences of RESP and SBP on RR in obesity. This finding indicates that the indirect connection between RESP and HRV – cascade RESP→ < *SBP* < / →RR – is partially suppressed in obese group compared to controls. It could reflect the initial impairment of cardiac chronotropic baroreflex function in this group – the observation found in previous studies by a decreased baroreflex sensitivity (Honzikova et al., 2006; Lazarova et al., 2009).

**FIGURE 3:**
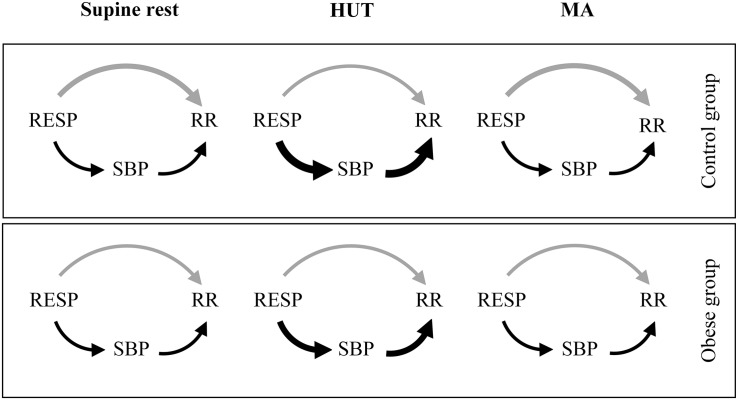
Proposed causal interaction models of RSA mechanisms during supine rest, HUT, and MA. The arrow thickness reflects the strength of the causal coupling in the given direction, with changes in thickness corresponding to statistically significant variations between groups or conditions. The gray arrow represents the direct effects of RESP on RR (U_RESP__→__RR_; strength of non-baroreflex RSA mechanisms) and the black arrows represent the effects of RESP on RR mediated through SBP (R_RESP,SBP__→__RR_; strength of baroreflex RSA mechanism).

The observed differences in U_RESP__→__RR_ could be also related to ventilatory pattern differences (Javorka et al., 2018). Therefore, we also measured tidal volume and respiratory rate from a calibrated RESP signal. Significantly higher tidal volume (*P* ≤ 0.036) and no significant differences in respiratory rate (*P* ≥ 0.129) were found in the obese patients compared to the control group. These differences – being mostly in favor of stronger respiratory influence on HRV – cannot be responsible for the observed between-group differences in the unique TE from RESP to RR. It should be noted that tidal volume reflects the amplitude of the respiratory input while information transfer reflects the involvement of the RSA-related mechanisms; therefore, the increased tidal volume (stronger input) together with the decreased information transfer (weaker link) could balance each other, possibly contributing to explain the preserved RSA magnitude found in obese patients across all experimental conditions. Taken together, our results indicate a slightly decreased parasympathetic HRV influence in young obese patients at rest. The results of the present study are summarized in the causal interaction models of RSA mechanisms during supine rest, HUT, and MA separately for healthy and obese adolescents and young adults (Figure 3).

Importantly, between-groups differences in PID parameters were not detectable when HF oscillations were removed and we analyzed the cardiovascular and respiratory time series on scale τ_2_ representing oscillations slower than those contained in the HF band. This indicates that observed subtle differences between groups reflected RESP-related oscillations.

From the clinical point of view, the results of our study point toward three important conclusions. Firstly, it is important to stress that while RSA magnitude (HF HRV) was not influenced by obesity, novel measures of the coupling strength between signals revealed subtle differences. We suggest that the coupling measures focused on the more detailed analysis of RSA mechanisms could be used in future for a detection of the subjects with impaired autonomic control not only associated with obesity. Secondly, significant differences between groups (obese vs. controls) were revealed mostly at stress conditions (orthostasis) pointing toward an importance of ANS testing during different physiological states (not only at rest). Lastly, we suggest that the analysis of interconnections between physiological signals can improve our understanding of the mechanisms underlying the oscillations. In our case, HF HRV (RSA) oscillations origin included both baroreflex and non-baroreflex mechanisms. The better understanding of the HRV mechanisms can improve the interpretability of the HRV analysis results.

## Conclusion

We conclude that the RSA magnitude and its responsiveness to physical and cognitive stress are well preserved in young obese subjects. However, the information domain analysis of cardiovascular and cardiorespiratory interactions contributing to the origin of RSA revealed subtle differences mostly during orthostasis pointing toward evidence of an initial parasympathetic nervous system impairment.

## Data Availability Statement

The datasets generated for this study are available on written request to the corresponding author JK (jana.krohova@uniba.sk).

## Ethics Statement

The studies involving human participants were reviewed and approved by the Ethics Committee of Jessenius Faculty of Medicine, Comenius University. Written informed consent to participate in this study was provided by the participants’ legal guardian/next of kin.

## Author Contributions

MJ, MC, and LF designed the study. MC and DC arranged for the probands participation. ZT, BC, NM, JK, and RW performed the measurements. JK and BC analyzed the data. MJ, JK, and LF wrote the manuscript. MJ, JK, LF, and RP contributed to the interpretation of the results. AB and MC helped supervise the project. All authors reviewed the manuscript.

## Conflict of Interest

The authors declare that the research was conducted in the absence of any commercial or financial relationships that could be construed as a potential conflict of interest.
